# Characteristic of clinical trials related to traumatic brain injury registered on ClinicalTrials.gov over the past two decades (2004–2023)

**DOI:** 10.3389/fmed.2024.1435762

**Published:** 2024-09-16

**Authors:** Ruili Guo, Qingya Yang, Xuan Zhou, Shining Li, Yao Liu

**Affiliations:** The First Affiliated Hospital of Ningbo University, Ningbo, China

**Keywords:** traumatic brain injury, clinical trial, ClinicalTrials.gov, study design, early discontinuation, therapy category

## Abstract

**Objective:**

The aim of this report is to provide a comprehensive overview of clinical trials and protocols related to traumatic brain injury over the past two decades.

**Methods:**

We collected information on clinical trials related to traumatic brain injury (TBI) from the ClinicalTrials.gov database, identified key categorical variables, and assessed their characteristics.

**Results:**

A total of 367 TBI-related trials were identified for analysis. All identified trials were interventional clinical trials. Most trials were small-scale, with 75.2% enrolling 1–100 participants, and only about 20% were funded by industry or the National Institutes of Health (NIH). In most trials, participants were gender-neutral (96.5%), and the primary age group was adults and older adults (56.9%). Of all identified TBI trials, 78.2% were randomized, and 69.4% were blinded. Additionally, the primary purpose of 297 trials (80.9%) was treatment, with drug therapy as the most common intervention. A total of 153 trials (41.7%) were completed; however, only 58 trials submitted results to the registry. Furthermore, 81 trials (22.1%) were discontinued early, primarily due to recruitment problems. Clinical trials started between 2004 and 2013 reported a higher proportion of results compared with those started between 2014 and 2023 (35.1% vs. 11.1%, *p* < 0.001). In addition, between 2014 and 2023, there was an increase in trials for diagnostic purposes (2.4% vs. 6.5%, *p* < 0.001).

**Conclusion:**

Based on the data collected from the ClinicalTrials.gov, our study reveals that most clinical trials related to TBI focus on drug-related treatments, underreporting remains a significant concern, and greater emphasis should be placed on improving the publication and dissemination of clinical trial results.

## 1 Introduction

Traumatic brain injury is a major cause of death and disability worldwide (1, 2). Most patients will have short-term or long-term cognitive, physical, and psychological dysfunction (3, 4). Sixty-nine million individuals are estimated to suffer TBI from all causes each year (5). In addition, the large expenditure on the clinical treatment of most TBI patients and the associated socioeconomic problems impose a heavy burden on the healthcare system, already making it a serious public health problem (6). For the clinical management of TBI, surgical treatment in the acute phase is mainly by removing mass lesions and decompressive craniectomy to prevent further propagation of the initial insult (7). Internal medicine mainly focuses on symptomatic treatment such as hemostasis, infection prophylaxis, reduce cerebral edema, reduction of intracranial pressure (8, 9). In the past few decades, although reliable evidence has been found for measures that can exert neuroprotective effects in TBI animal models, the translation of such neuroprotective strategies to human injury has been disappointing (10, 11). Most drugs did not pass phase 2 clinical trials, and all phase 3 clinical trials failed, there is currently no clinically effective treatment to improve functional recovery after TBI (12).

A better understanding of the current characteristics of clinical trials related to traumatic brain injury has important implications for improving clinical trial design and identifying overlooked areas of research. Therefore, the aim of this report is to provide a comprehensive overview of clinical trials and protocols related to TBI over the past two decades.

## 2 Methods

### 2.1 Data collection

ClinicalTrials.gov is the world’s most comprehensive clinical trial registry, sharing information on clinical research worldwide (13). Sponsors and researchers entered data through a web-based data entry system (14).

To generate a dataset of interventional clinical trials for traumatic brain injury, data were downloaded from the ClinicalTrials.gov website on Jan 24, 2024, based on the following filters:

Condition or disease: traumatic brain injury; brain injuries traumatic; Brain Trauma; traumatic brain injuries; brain injury traumatic; Traumatic encephalopathy; traumatic brain damage; Traumatic Encephalopathies; brain; Cerebral; human brain; brain injury; Brain Injuries; Brain damage; Acquired brain injury; injury brain; cerebral injury; cerebral damage; injury; Trauma; injuries; Traumatic Injury; Wound; TBI; tbi; brain injuries traumatic; Traumatic Brain Injury; Brain Trauma; traumatic brain injuries; brain injury traumatic; Traumatic encephalopathy; traumatic brain damage; Traumatic Encephalopathies.

Study Phase: Early Phase 1, Phase 1, Phase 2, Phase 3, Phase 4.

All available results were downloaded as XML files. Subsequently, all data was imported into Excel tables to facilitate further data selection and extraction.

### 2.2 Selection criteria and data extraction

The inclusion criteria for the trials assessed in this study were as follows: (1) the clinical indication must be related to traumatic brain injury; (2) trials were commenced between January 1, 2004, and December 31, 2023. The following data for each eligible trial were extracted independently by two investigators: start date, trial status, study design, intervention, types of interventions, characteristics of participants, sample size, the primary purpose of the trial, funding sources, and study results. The data was subsequently verified by a third researcher.

### 2.3 Statistical analysis

Descriptive statistics were performed on the collected data with frequency and percentage. The year 2013, the midpoint of the 2004–2023 period, was chosen as the cutoff to compare the characteristics of trials.

The chi-squared test was applied to assess heterogeneity in the proportion of funding sources, the intervention type, blinding, primary purpose, enrollment, and phase. All analyses were conducted using SPSS version 18.0 (SPSS Inc., Chicago, IL, United States), and a *p* value <0.05 was considered statistically significant.

## 3 Results

### 3.1 Distribution of clinical trials related to TBI

**Figure 1:**
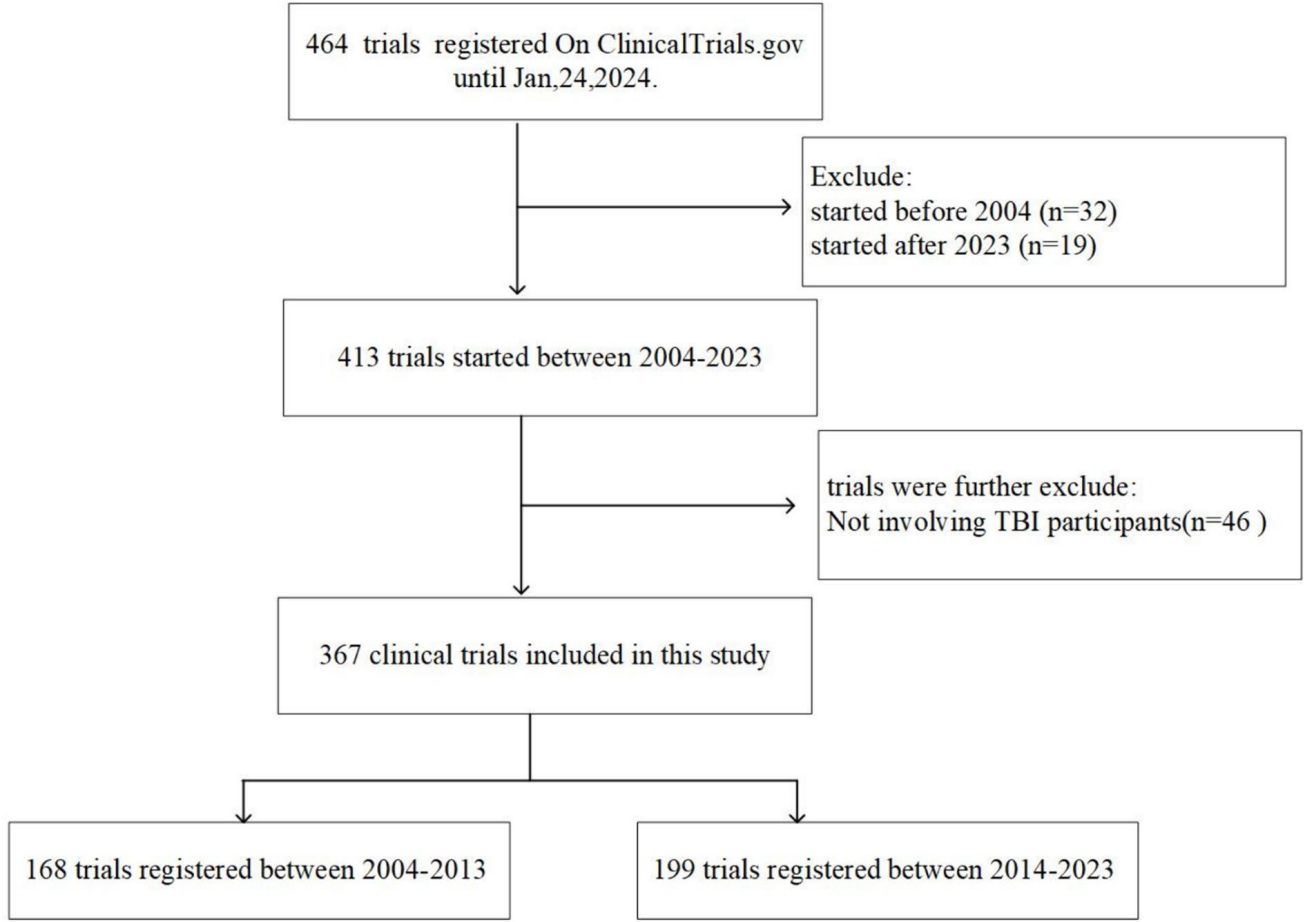
Flow chart of trial selection.

A total of 464 registered clinical trials were retrieved from the Clinicaltrials.gov database. Of these, 51 trials initiated before 2004 or after 2023, along with those including non-TBI participants (*n* = 46), were excluded. We thus identified 367 clinical trials associated with TBI that were initiated between January 1, 2004 and December 31, 2023. Figure 1 shows the selection procedure of this study.

**Figure 2:**
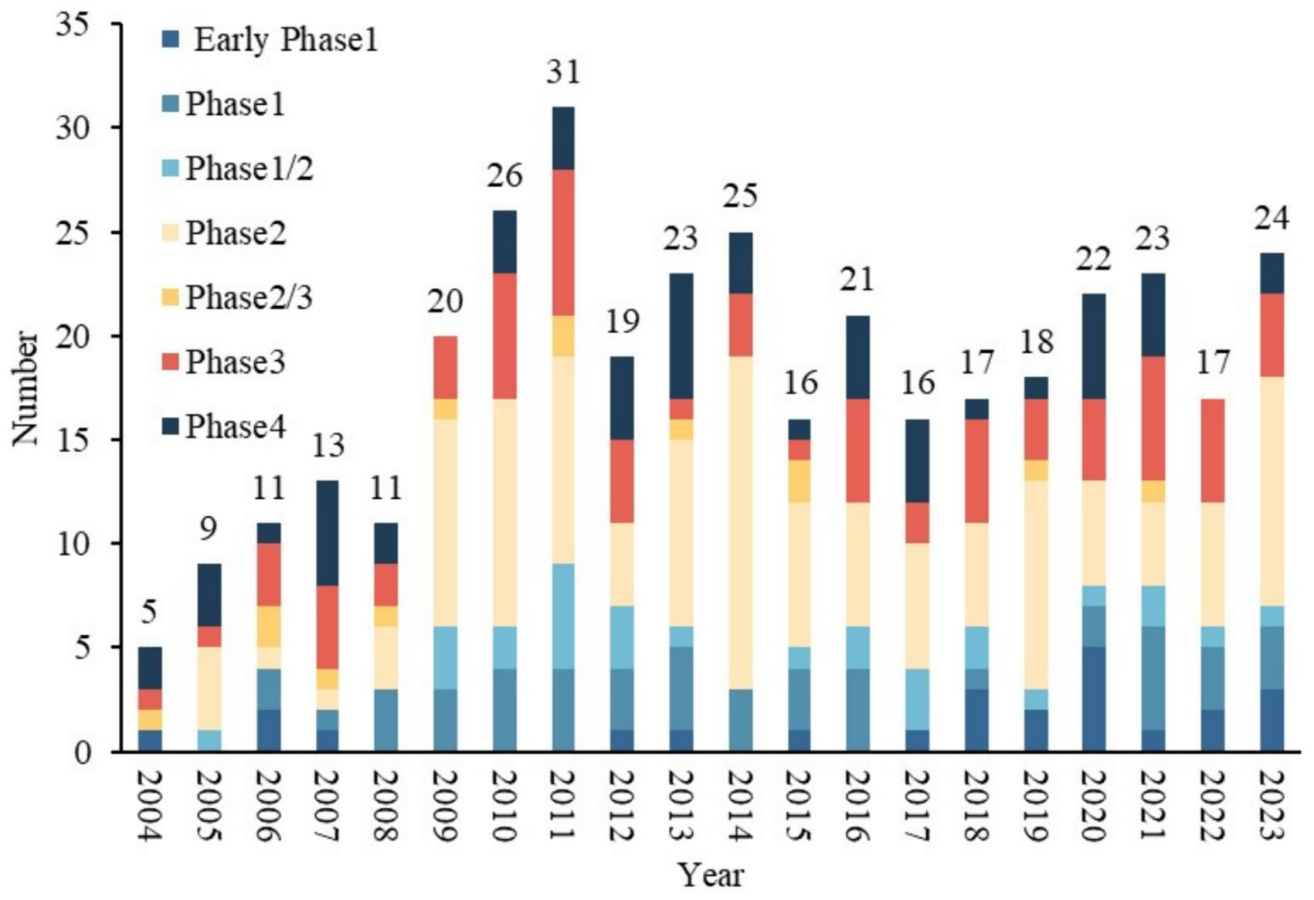
Distribution of clinical trials related to TBI according to the registered year.

The distribution of trials by registration year is summarized in Figure 2. Overall, the number of TBI trials showed an increasing trend after 2004, peaking at 31 trials in 2011. In addition, a significant increase was observed in 2009, the number of trials increased from 11 in 2008 to 20 in 2009, representing an increase of 81.5%. The number of trials decreased in 2012 and has since remained stable at approximately 20 per year. Among the five clinical trial phases (early phase I–IV) conducted from 2004 to 2023, phase II trials showed the greatest growth, followed by phase III, with AAGRs of 51.5 and 45.4%, respectively.

### 3.2 General characteristics of TBI trials

The general characteristics of identified trials between 2004–2013 and 2014–2023 are shown in Table 1. Most trials included both males and females participants (*n* = 354, 96.5%). Participants were divided into three age groups: children (from birth to 17 years old), adults (18 to 64 years old), and older adults (65 years and above). The largest age group among TBI trial participants was adults and older adults (*n* = 209, 56.9%) 0.4.1% of the TBI trials included only child participants, while 21.3% included only adult participants. 75.2% of the TBI trials were small studies involving 1–100 participants. In terms of funding, the most common source was other (*n* = 296, 80.7%), followed by industry (*n* = 60, 16.3%) and NIH (*n* = 11, 3.0%).

**Table 1:** General characteristics of TBI trials (*n* = 367).

Characteristics	Total *N* (%)	2004–2013 *N* (%)	2014–2023 *N* (%)	*χ*^2^	*p*-value
Gender					
All	354 (96.5%)	163 (97.0%)	191 (96.0%)	0.291	0.403
Male	13 (3.5%)	5 (3.0%)	8 (4.0%)		
Age group					
Child	15 (4.1%)	9 (5.4%)	6 (3.0%)	6.540	0.288
Adult	78 (21.3%)	32 (19.0%)	46 (23.1%)		
Child + Adult	24 (6.5%)	12 (7.1%)	12 (6.0%)		
Adult + Older Adult	209 (56.9%)	90 (53.6%)	119 (59.8%)		
Child + Adult + Older Adult	41 (11.2%)	25 (14.9%)	16 (8.0%)		
Enrollment					
0–100	274 (75.2%)	129 (76.8%)	147 (73.9%)	0.594	0.352
101–200	42 (51.9%)	17 (10.1%)	25 (12.6%)		
>200	49 (17.1%)	22 (13.1%)	27 (13.6%)		
Funded by					
Industry	60 (16.3%)	31 (18.5%)	29 (14.6%)	1.455	0.483
NIH	11 (3.0%)	6 (3.6%)	5 (2.5%)		
Other	296 (80.7%)	131 (35.7%)	165 (45.0%)		
Interventions*
Drug	253 (68.9%)	118 (32.4%)	134 (36.5%)	6.325	0.621
Device	38 (10.4%)	16 (4.4%)	22 (6.0%)		
Biological	21 (5.7%)	10 (2.7%)	11 (3.0%)		
Behavioral	30 (8.2%)	15 (4.1%)	15 (4.1%)		
Procedure	14 (3.8%)	7 (1.9%)	7 (1.9%)		
Combination Product	8 (2.2%)	3 (0.8%)	5 (1.4%)		
Dietary Supplement	11 (3.0%)	3 (0.8%)	8 (2.2%)		
Radiation	3 (0.8%)	0 (0.0%)	3 (0.8%)		
Other	55 (15.0%)	20 (5.4%)	35 (9.5%)		
Status					
Proceeding	79 (21.5%)	1 (0.6%)	78 (39.2%)	88.564	<0.001
Completed	153 (41.7%)	93 (55.4%)	60 (30.3%)		
Unknown status	54 (14.7%)	22 (13.1%)	32 (16.1%)		
Early discontinuation	81 (22.1%)	52 (31.0%)	29 (14.9%)		
Result					
No	286 (22.1%)	109 (64.9%)	177 (88.9%)	30.669	<0.001
Has	81 (77.9%)	59 (35.1%)	22 (11.1%)		

*Some included trials have more than one interventions.

Of the 367 identified TBI trials, 81 (22.1%) were discontinued early, including 49 terminated, 2 suspended, and 30 withdrawn (Table 1). A total of 153 trials (41.7%) were completed; however, only 81 submitted results to the registry. Furthermore, the percentage of trials reporting results decreased from 35.1% in 2004–2013 to 11.1% in 2014–2023 (*p* < 0.001). More trials are proceeding between 2014 and 2023 (0.6% vs. 39.2%, *p* < 0.001).

### 3.3 Design characteristic of TBI trials registered in ClinicalTrials.gov

**Figure 3:**
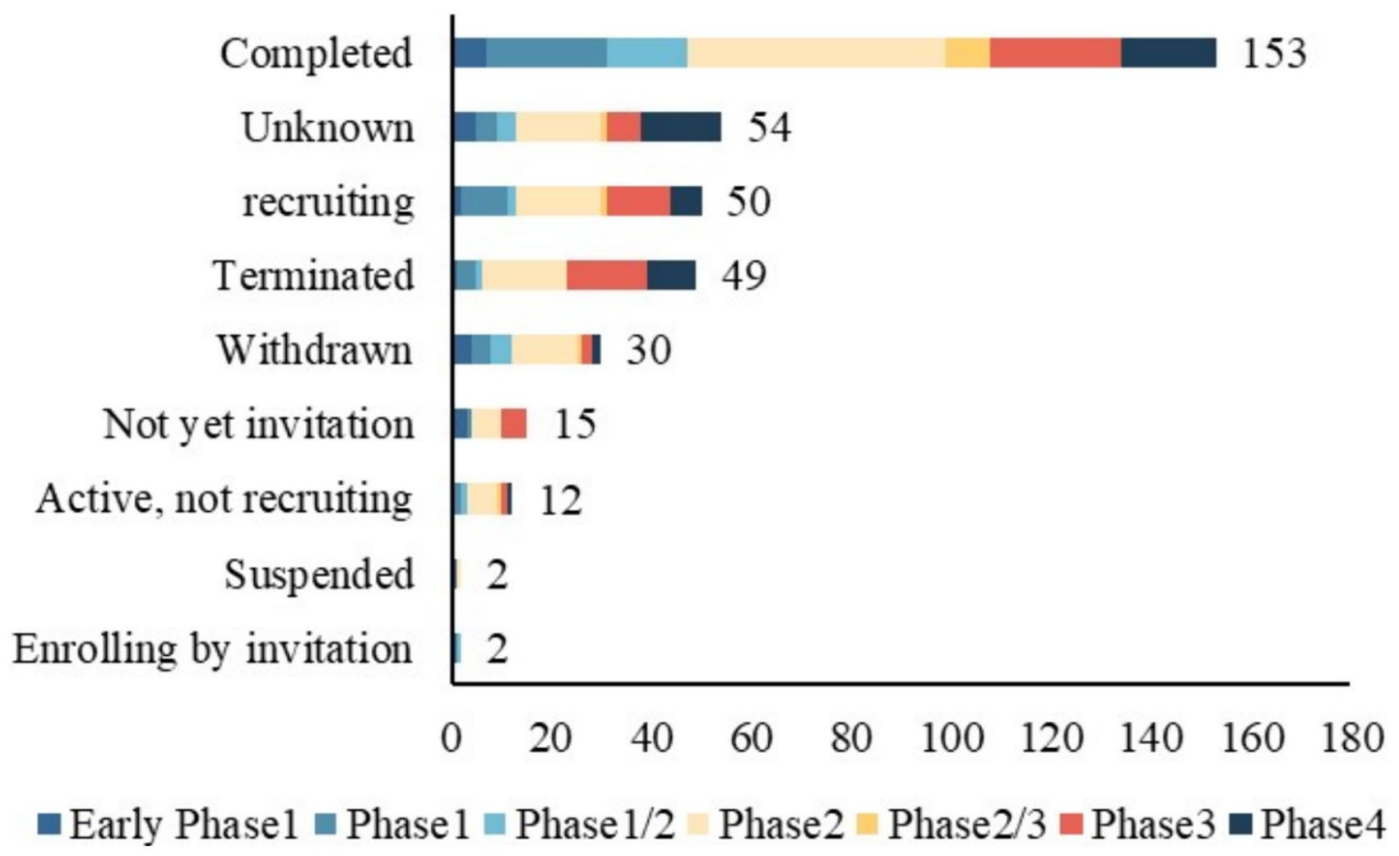
Distribution of study phase and status for clinical trials.

Table 2 presents the design characteristics of TBI trials conducted between 2004–2013 and 2014–2023. All included trials were interventional clinical trials. Of the 367 trials, phase 2 accounted for the largest proportion of 35.1%. The distribution of clinical trials by study phase and status is shown in Figure 3. Additionally, 78.2% were randomized. 81.2% of the trials included control groups and the vast majority (71.1%) performed parallel assignments. Notably, 69.4% of the studies were blinded. The most frequently blinded subjects were participants (*n* = 217), followed by investigators (*n* = 162), outcome assessors (*n* = 167), and care providers (*n* = 135). Excluding clinical trials that did not specify a primary purpose, the primary purpose of TBI trials involved seven categories, of which treatment purpose was the most common type (*n* = 297, 80.9%). The other categories included Health Services Research (*n* = 2, 0.5%), Supportive Care (*n* = 8, 2.2%), Basic Science (*n* = 9, 2.5%), Diagnostic (*n* = 17, 4.6%), Prevention (*n* = 25, 6.8%), Other (*n* = 5, 1.4%). The proportion of intervention trials with diagnosis as the primary purpose increased from 2.4 to 6.5% during the two periods (*p* < 0.001).

**Table 2:** Design characteristics of TBI trials registered in ClinicalTrials.gov (*n* = 367).

Characteristics	Total *N* (%)	2004–2013 *N* (%)	2014–2023 *N* (%)	*χ*^2^	*p*-value
Phases				10.324	0.076
Early phase 1	24 (6.5%)	6 (3.6%)	18 (9.0%)		
Phase 1	48 (13.1%)	24 (14.3%)	24 (12.1%)		
Phase 1|Phase 2	29 (7.9%)	15 (8.9%)	14 (7.0%)		
Phase 2	129 (35.1%)	53 (31.5%)	76 (38.2%)		
Phase 2|Phase 3	13 (3.5%)	9 (5.4%)	4 (2.0%)		
Phase 3	70 (19.1%)	32 (19.0%)	38 (19.1%)		
Phase 4	54 (14.7%)	29 (17.3%)	25 (12.6%)		
Allocation				0.509	0.482
Randomized	287 (78.2%)	130 (77.4%)	157 (78.9%)		
Non-Randomized	35 (9.5%)	18 (10.7%)	17 (8.5%)		
Allocation: N/A	45 (12.3%)	20 (11.9%)	25 (12.6%)		
Intervention model				12.092	0.011
Single group assignment	69 (18.85)	42 (25.0%)	27 (13.6%)		
Crossover assignment	27 (7.4%)	11 (6.5%)	16 (8.0%)		
Parallel assignment	261 (71.1%)	109 (64.9%)	152 (76.4%)		
Sequential assignment	4 (1.1%)	1 (0.6%)	3 (1.5%)		
Factorial assignment	6 (1.6%)	5 (3.0%)	1 (0.5%)		
Masking				6.777	0.072
Single	50 (13.6%)	23 (13.7%)	27 (13.6%)		
Double	65 (17.7%)	21 (12.5%)	44 (22.1%)		
Triple	37 (10.1%)	17 (10.1%)	20 (10.1%)		
Quadruple	99 (27.0%)	51 (30.4%)	48 (24.1%)		
None (open label)	113 (30.8%)	54 (32.1%)	59 (29.6%)		
Not provided	3 (0.8%)	2 (1.2%)	1.0 (0.5%)		
Primary purpose				9.527	0.036
Health Services Research	2 (0.5%)	2 (1.2%)	0 (0.0%)		
Supportive Care	8 (2.2%)	3 (1.8%)	5 (2.5%)		
Basic Science	9 (2.5%)	3 (1.8%)	6 (3.0%)		
Diagnostic	17 (4.6%)	4 (2.4%)	13 (6.5%)		
Prevention	25 (6.8%)	12 (7.1%)	13 (6.5%)		
Treatment	297 (80.9%)	140 (83.3%)	157 (78.9%)		
Other	5 (1.4%)	1 (0.6%)	4 (2.0%)		
Not provided	4 (1.1%)	3 (1.8%)	1 (0.5%)		

### 3.4 Interventions in TBI trials

The interventions in TBI-related clinical trials included single-type interventions (*n* = 303) and combinations of different intervention types (*n* = 64) (Table 3). Single-intervention trials were categorized into nine types, drug therapy was the most common type (*n* = 214, 58.3%). In addition, the category with the largest proportion of clinical trials combining the two interventions was also drug-related.

**Table 3:** Interventions in TBI trials.

Items	Detail	Number	Percent
Single Intervention/treatment	Drug	214	58.3
	Device	25	6.8
	Biological	14	3.8
	Behavioral	22	6.0
	Other	13	3.5
	Procedure	5	1.4
	Combination Product	3	0.8
	Dietary Supplement	6	1.6
	Radiation	1	0.3
	Sub Total	303	82.6
Combination of two interventions/treatment	Dietary Supplement + Other	2	0.5
	Behavioral + Device	1	0.3
	Behavioral + Drug	4	1.1
	Behavioral + Other	3	0.8
	Biological + Drug	2	0.5
	Biological + Other	4	1.1
	Biological + Procedure	1	0.3
	Combination Product + Device	1	0.3
	Combination Product + Other	1	0.3
	Device + Drug	4	1.1
	Device + Other	6	1.6
	Procedure + Other	4	1.1
	Drug + Other	22	6.0
	Drug + Procedure	4	1.1
	Drug + Dietary Supplement	2	0.5
	Dietary + Genetic	1	0.3
	Radiation + Drug	1	0.3
	Radiation + Device + Genetic	1	0.3
	Sub Total	64	17.4

### 3.5 Therapy category analysis

**Figure 4:**
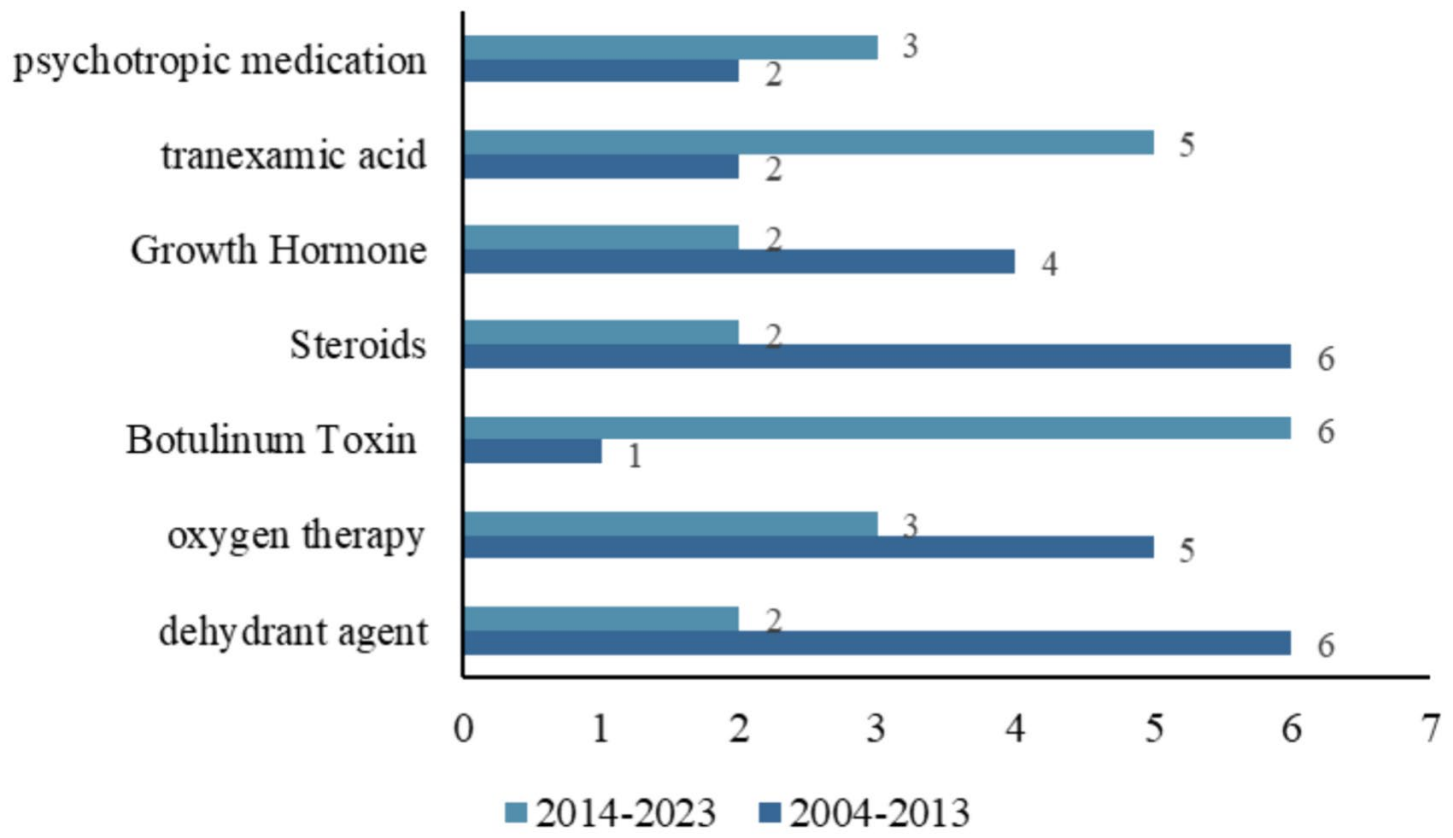
Distribution of the common drugs studied in all TBI-related clinical trials between two temporal subsets (2004–2013 and 2014–2023).

We further analyzed the drugs included in clinical trials with a treatment purpose. Among the 297 trials aimed at treatment, divided into single interventions and combinations of two interventions. A total of 199 clinical trials involved drug-related interventions, details are shown in Supplementary Table S1. Figure 4 shows the trends of common drugs in TBI-related clinical trials in the two periods of 2004–2013 and 2014–2023. Dehydrant agents, oxygen therapy, botulinum Toxin, sex hormones, Growth Hormone, tranexamic acid, and psychotropic medication were the most commonly studied drugs in the trials. From 2014 to 2023, the number of trials focused on dehydrant agents and Growth Hormones increased rapidly, while trials on botulinum toxin decreased significantly.

We also investigated 17 clinical trials with diagnostic purposes, where the main interventions included drug, radiation, and device, all of which had more clinical trials conducted between 2014–2023 than between 2004–2013, with most of the drugs being used to assist in imaging. Interestingly, a clinical trial involving computerized clinical decision support was conducted between 2004 and 2013, but no clinical trials of such computer-assisted procedures were conducted in the following decade.

### 3.6 Characteristics associated with early trial discontinuation

A total of 81 clinical trials were prematurely discontinued. The majority of clinical trials were withdrawn (*n* = 30), terminated (*n* = 49), or suspended (*n* = 2). These discontinuations occurred primarily during phase 2 (*n* = 31), followed by phase 3 (*n* = 18) and phase 4 (*n* = 12). Mainly due to recruitment problems (*n* = 27), funding problems (*n* = 7), lack of efficacy (*n* = 8), the principal investigator or the investigator leaving the institution (*n* = 4), etc. The COVID-19 pandemic has undoubtedly had a significant impact on clinical trial programs. It is encouraging that only six TBI trials listed on the ClinicalTrials.gov were withdrawn due to COVID-19. Some trials were prematurely discontinued for more than one reason. Details are provided in Table 4.

**Table 4:** Characteristics associated with early trial discontinuation.

Items	Detail	Number	Phases	Reasons for trial termination
Status	Withdrawn	30	Early Phase 1 (*n* = 4) Phase 1 (*n* = 4) Phase1/Phase2 (*n* = 4) Phase2 (*n* = 13) Phase2/Phase3 (*n* = 1) Phase 3 (*n* = 2) Phase 4 (*n* = 2)	Recruitment problems (*n* = 5) Futility (*n* = 2) PI left the institution (*n* = 2) Test device not approved to be used (*n* = 1) Funding problems (*n* = 2) No reason (*n* = 8) COVID (*n* = 2) Never started (*n* = 3) The manufacturer has stopped making the medicine (*n* = 2) New alternate study (*n* = 3)
	Terminated	49	Early Phase 1 (*n* = 1) Phase 1 (*n* = 4) Phase1/Phase2 (*n* = 1) Phase2 (*n* = 17) Phase 3 (*n* = 16) Phase 4 (*n* = 10)	Recruitment problems (*n* = 22) Futility (*n* = 6) Investigators left the institution (*n* = 2) Reprioritization of company activities (*n* = 6) Funding problems (*n* = 5) No reason (*n* = 8) COVID (*n* = 3) Administrative (*n* = 1)
	Suspended	2	Early Phase 1 (*n* = 1) Phase2 (*n* = 1)	COVID (*n* = 1) No reason (*n* = 1)

### 3.7 Characteristics associated with reporting results in the registry

Table 5 provides a summary of the characteristics of the completed trials (*n* = 153). Only 58 trials submitted results to the registry. A chi-squared test was conducted to compare categorical variables. Drug trials and randomized trials reported a higher proportion of results in ClinicalTrials.gov compared to non-drug trials and non-randomized trials.

**Table 5:** Characteristics associated with reporting results in the registry.

	Has results (*n* = 58)	No results available (*n* = 95)	*χ*^2^	*p*-value
Enrollment			0.004	0.554
0–100	43	70		
>100	15	25		
Phase			0.825	0.254
Phase1–3	49	85		
Phase 4	9	10		
Intervention			18.361	<0.001
Non-drug	45	40		
Drug	13	55		
Blinding			0.382	0.334
Non	15	29		
Single-quadruple	43	66		
Primary Purpose			2.792	0.067
Treatment	42	60		
Non-treatment	13	35		
Allocation			8.419	0.015
N/A	8	9		
Non-randomized	18	13		
Randomized	32	73		

## 4 Discussion

The purpose of this study was to provide an overview of TBI-related clinical trials registered in the ClinicalTrials.gov database. To our knowledge, this is the first comprehensive assessment of clinical trial characteristics associated with TBI. Our results suggest that TBI-related trials tend to have more participants in the adult and older age groups, and are typically not funded by industry or the NIH. Additionally, 75.2% of TBI trials were small-scale studies involving no more than 100 participants.

In terms of study design, the majority of the trials employed random allocation (78.2%) and parallel assignment models (71.1%). Furthermore, 68.4% of the studies were blinded. It has been reported that trials Randomization essentially eliminates threats to study validity such as reverse causation and selection bias, and significantly mitigates the influence of confounding factors. Blinding may exaggerate the benefits of the intervention (15). Ensuring that the trial provides a reliable treatment comparison by appropriate randomization and blinding is an essential element of designing a valuable clinical trial (16). Randomization essentially eliminates the threats of reverse causation and selection bias to study validity, and, significantly mitigates the influence of confusion. In addition, 6.8% of the trials focused on prevention. 4.6% of the trials belonged to the category of diagnostic. Moreover, very few trials belonged to the category of health services research and supportive care.

The present results indicate that 22.1% of TBI trials were discontinued early. Recruitment problem was the most common reason for discontinuation. Failure to complete a TBI clinical trial should prompt researchers to optimize enrollment strategies by identifying barriers in trial design. From the patients’ perspective, network media should be used to expand the audience and improve public participation. Additionally, eliminating enrollment concerns through an enhanced informed consent process should be considered. From the investigator’s perspective, misestimating expected enrollment, along with overly stringent eligibility criteria or time constraints, can lead to recruitment difficulties. These issues should be thoroughly addressed before initiating clinical trials.

We also examined the proportion of reporting study results. The findings revealed that 62.1% of completed trials did not submit their results to the registry. The Food and Drug Administration Amendments Act and Final Rule did not mandate that all studies report their results to the registry, which may explain explaining the lower rate of reporting in the TBI field (17). Prompt reporting and publication of results to the registry is crucial. The bias created by selective reporting leads to wasted research resources and is detrimental to clinical practice (18). Furthermore, our results showed that among completed clinical trials, a higher proportion of trials reporting results with the intervention were drug than non-drug.

Registration of clinical trials has improved significantly since the International Committee of Medical Journal Editors (ICMJE) proposed a trial for publication only if it has been registered before the enrollment of the first patient (19, 20). The results of the present study showed that the number of trials started in 2014–2023 was greater than in 2004–2013. This may indicate a growing focus on TBI. TBI is a highly heterogeneous disease, the treatment strategy is divided into acute neuroprotective therapy and subacute neurorestorative therapy, the former strategy focuses on reducing secondary injury and nerve cell death, and reducing the size of the lesion; unlike neuroprotective, restorative therapy aims to reshape brain tissue, not just solely against cell death or lesion volume (21). Considering the heterogeneity of the pathology, a single intervention may not address all pathological mechanisms of action.

Drug-related therapy has always been a hot topic in clinical trials related to TBI. We found that 17.4% of clinical trial interventions involved multiple categories beyond a single intervention. The number of clinical trials involving devices is second only to those involving drugs. Combining multiple interventions may better address the complex pathological processes following TBI. Although many potentially effective therapies have been identified in preclinical studies over the past 20 years, these results have not been replicated in clinical trials, and no drug has shown clear benefits for functional recovery. This discrepancy may be due to biological differences between humans and rodents (22). How to translate the results of preclinical research in the field of TBI into practical clinical application is still a question worth exploring. The current approach is paramount in reducing morbidity and mortality among populations worldwide. Based on clinical or physiological manifestations, particular forms of symptomatic therapy are used to reduce secondary injury. Given the complexity of the pathophysiology of TBI and its high heterogeneity, a thorough analysis of this topic is worth exploring in the future.

Although the diagnostic products involved in the interventional clinical trials discussed in this study have not yet amassed sufficient evidence for marketing approval, ongoing clinical trials are aimed at verifying the validity of TBI diagnosis through blood tests, specialized brain imaging, and portable imaging devices. Notably, a prospective cohort study has yielded positive results over the past two decades, leading to the United States Food and Drug Administration’s (FDA) approval of the TBI Indicator^™^ (BTI^™^) kit for TBI diagnosis in 2018 (23). While this is the only product approved for clinical use between 2004–2023, in 2024, a rapid handheld objective blood test product called i-STAT TBI Plasma test was approved for marketing by FDA. The test requires the use of the Abbott i-STAT TBI Plasma cartridge and the Abbott i-STAT Alinity analyzer to provide results (24). Both assays use ubiquitin C-terminal hydrolase-L1 and glial fibrillary acidic protein as tandem biomarkers (25). Other potential diagnostic tests for TBI are still under investigation.

Although this study is the first to present the specific characteristics of TBI-related trials on ClinicalTrials.gov over the past two decades, the limitations of our study should be clarified. First, ClinicalTrials.gov is not the only clinical trial registry. Although it contains the highest number of registered trials, it may not include all clinical trials. Researchers can use other global registration platforms to meet the mandatory registration guidelines advocated by ICMJE, trials registered in non-ClinicalTrials.gov was not included in this study, therefore, the conclusions of this study should be interpreted with caution. Secondly, the data in ClinicalTrials.gov are derived from self-reports from trial sponsors or investigators, and not all information is updated regularly, which may limit analysis. Finally, owing to the multiple complex mechanisms of drugs, we did not classify all drugs in clinical trials.

## 5 Conclusion

In conclusion, this study is the first to present the specific characteristics of registered TBI-related clinical trials over the past two decades. Our study results indicated that these trials were dominated by small studies. Most TBI-related clinical trials focus on drug-related treatments. Most trials lacked the availability of results, underreporting is a concern, and more emphasis should be placed on improving the publication and dissemination of clinical trial results.

## Data Availability

The raw data supporting the conclusions of this article will be made available by the authors, without undue reservation.
