# Supplementary material for: Characteristic of clinical trials related to traumatic brain injury registered on ClinicalTrials.gov over the past two decades (2004–2023)
Source: Front Med (Lausanne). 2024 Sep 16;11:1435762. doi: 10.3389/fmed.2024.1435762 (PMC11439763; doi:10.3389/fmed.2024.1435762)
Supplement: Supplementary file 1 [file Table_1.DOCX]

**Supplementary Table 1：Clinical trials for therapeutic purposes related to drugs**

| NCT Number | Study Title | Interventions | Study Status | Start Date | Phases |
| --- | --- | --- | --- | --- | --- |
| NCT00878631 | Feasibility Trial of Traumatic Brain Injured Patients Randomized in the Prehospital Setting to Either Hypertonic Saline and Dextran Versus Normal Saline | DRUG: hypertonic saline mixed with dextran\|DRUG: Normal Saline | COMPLETED | 2004/9/1 | PHASE2\|PHASE3 |
| NCT00205491 | Pharmacological Intervention in Depression After Traumatic Brain Injury | DRUG: Venlafaxine | COMPLETED | 2004/10/1 | PHASE4 |
| NCT00123591 | Safety and Preliminary Efficacy of Recombinant Activated Factor VII in Subjects With Traumatic Brain Injury | DRUG: activated recombinant human factor VII | COMPLETED | 2005/1/1 | PHASE2 |
| NCT02130674 | Optimized Therapy in Severe Traumatic Brain Injured Patients | DRUG: Dipeptiven | COMPLETED | 2005/1/1 | PHASE4 |
| NCT00162916 | Antidepressant Maintenance in Traumatic Brain Injury | DRUG: citalopram\|DRUG: Placebo | UNKNOWN | 2005/5/1 | PHASE4 |
| NCT00219869 | Galantamine in the Treatment of Post-Traumatic Headache | DRUG: Galantamine | UNKNOWN | 2005/7/1 | PHASE4 |
| NCT00233090 | Post-Traumatic Brain Injury (Post-TBI) Fatigue and Its Treatment | DRUG: Modafinil\|DRUG: Placebo | TERMINATED | 2005/7/1 | PHASE2 |
| NCT00174980 | Study of Oxycyte in Severe Closed Head Injury | DRUG: perfluorocarbon emulsion (Oxycyte) infusion | COMPLETED | 2005/9/1 | PHASE2 |
| NCT00254722 | Safety of Autologous Stem Cell Treatment for Traumatic Brain Injury in Children | DRUG: Autologous bone marrow precursor cell harvest and transplant | COMPLETED | 2006/4/1 | PHASE1 |
| NCT00313716 | Effects of Erythropoietin on Cerebral Vascular Dysfunction and Anemia in Traumatic Brain Injury | DRUG: recombinant human erythropoietin, rhEpo\|OTHER: placebo | COMPLETED | 2006/4/1 | PHASE2\|PHASE3 |
| NCT00316004 | Hypertonic Resuscitation Following Severe Traumatic Brain Injury (TBI) | DRUG: 7.5% Hypertonic Saline in 6% Dextran-70 (HSD)\|DRUG: 7.5% Hypertonic Saline (HS)\|DRUG: 0.9% Normal Saline (NS) | TERMINATED | 2006/5/1 | PHASE3 |
| NCT00336882 | Anaesthesia With Propofol Versus Midazolam : Effect on Oxidative Stress in the Brain of Head Trauma Patients | DRUG: Propofol\| DRUG: Midazolam | TERMINATED | 2006/6/1 | PHASE3 |
| NCT00487578 | Naratriptan for the Treatment of Post Traumatic Headache Associated With Cognitive Dysfunction | DRUG: naratriptan HCl | TERMINATED | 2006/10/1 | PHASE4 |
| NCT00375869 | Safety of Darbepoetin Alfa Treatment in Patients With Severe Traumatic Brain Injury | DRUG: Darbeopoetin\|DRUG: Normal Saline (Placebo) | COMPLETED | 2006/11/1 | PHASE2 |
| NCT00287157 | Pilot, Proof-of-Concept Study of Sublingual Tizanidine in Children With Chronic Traumatic Brain Injury (TBI) | DRUG: Sublingual Tizanidine HCl | COMPLETED | 2006/12/1 | PHASE1 |
| NCT00594503 | Hyperbaric Oxygen Therapy and SPECT Brain Imaging in Traumatic Brain Injury | DRUG: Hyperbaric oxygen therapy | UNKNOWN | 2007/1/1 | PHASE1 |
| NCT00462228 | Effect of Namenda on Short Term Memory and Attention in Patients With Mild to Moderate Traumatic Brain Injury | DRUG: Memantine\|DRUG: Placebo | TERMINATED | 2007/4/1 | PHASE4 |
| NCT00432263 | Treatment Of Adult Growth Hormone Deficiency After Traumatic Brain Injury. | DRUG: Genotropin (PN-180,307) Somatropin | WITHDRAWN | 2007/4/1 | PHASE4 |
| NCT00491192 | Normothermia in Patients With Acute Cerebral Damage | DRUG: Diclofenac | UNKNOWN | 2007/6/1 | PHASE4 |
| NCT00929045 | Growth Hormone and Insulin Growth Factor 1 Deficiencies in Children/Adolescents Following Traumatic Brain Injury: The Impact on Growth and Neuropsychological Development | DRUG: Growth Hormone Replacement | COMPLETED | 2007/7/1 | EARLY_PHASE1 |
| NCT00545662 | Study of Citicoline for the Treatment of Traumatic Brain Injury (COBRIT) | DRUG: Placebo\|DRUG: citicoline | TERMINATED | 2007/7/1 | PHASE3 |
| NCT00618436 | Assess Safety and Efficacy of Levetiracetam(LEV;Keppra)for Seizure Prevention | DRUG: Levetiracetam\|DRUG: Phenytoin | COMPLETED | 2007/8/1 | PHASE4 |
| NCT01814982 | An Efficacy, Safety, Pharmacokinetics, and Pharmacodynamics Study of JNJ-17299425 in Participants With Traumatic Brain Injury | DRUG: JNJ-17299425 | TERMINATED | 2007/8/1 | PHASE2 |
| NCT00555009 | Treatment Of Adult Growth Hormone Deficiency After Traumatic Brain Injury | DRUG: Genotropin\|DRUG: Placebo | TERMINATED | 2008/3/1 | PHASE4 |
| NCT01028339 | Mannitol vs HS to Treat ICHT After Severe TBI : Comparison on PtiO2 and Microdialysis Values | DRUG: Hypertonic saline\|DRUG: Mannitol | TERMINATED | 2008/7/1 | PHASE3 |
| NCT00766038 | Recombinant Human Growth Hormone During Rehabilitation From Traumatic Brain Injury. | DRUG: Recombinant human Growth Hormone\|DRUG: Placebo | COMPLETED | 2008/9/1 | PHASE2 |
| NCT00760734 | Hyperbaric Oxygen Therapy (HBOT) in Chronic Traumatic Brain Injury (TBI)/Post Concussion Syndrome (PCS) and TBI/Post-Traumatic Stress Disorder (PTSD) | DRUG: Low pressure hyperbaric oxygen therapy\|DRUG: Low pressure hyperbaric oxygen therapy | COMPLETED | 2008/9/1 | PHASE1 |
| NCT00755209 | Tranexamic Acid for Preventing Progressive Intracranial Haemorrhage in Traumatic Brain Injury | DRUG: tranexamic acid | COMPLETED | 2008/10/1 | PHASE3 |
| NCT00795587 | Comparison of 2 Doses of Mannitol on Post Traumatic Intracranial Hypertension and Cerebral Monitoring | DRUG: variation of mannitol dose | TERMINATED | 2008/10/1 | PHASE4 |
| NCT00556387 | Efficacy of Ketamine in Children With Severe Brain Injury for Brain Cell Protection | DRUG: saline\|DRUG: Ketamine | WITHDRAWN | 2008/12/1 | PHASE2 |
| NCT00727246 | CDP-Choline and Working Memory After TBI: A Neuroimaging Study | DRUG: CDP-Choline\|DRUG: Placebo | COMPLETED | 2009/3/1 | PHASE2 |
| NCT00735085 | A Phase 2a Dose Escalation Study With SLV334 in Patients With Traumatic Brain Injury. | DRUG: SLV334\|DRUG: SLV334\|DRUG: SLV334\|DRUG: SLV334\|DRUG: Placebo | TERMINATED | 2009/4/1 | PHASE2 |
| NCT00893789 | Study to Evaluate the Efficacy and Safety of Armodafinil as Treatment for Patients With Excessive Sleepiness Associated With Mild or Moderate Closed Traumatic Brain Injury | DRUG: Armodafinil\|DRUG: Armodafinil\|DRUG: Armodafinil\|OTHER: Placebo | TERMINATED | 2009/4/30 | PHASE3 |
| NCT00973674 | Resuscitative Endocrinology:Single-dose Clinical Uses for Estrogen-Traumatic Brain Injury | DRUG: Premarin IV\|DRUG: Placebo | COMPLETED | 2009/7/1 | PHASE2 |
| NCT00930202 | Study of the Safety and Efficacy of Conivaptan (Vaprisol庐) to Raise Serum Sodium Levels in Patients With Severe Traumatic Brain Injury | DRUG: Conivaptan (Vaprisol) | COMPLETED | 2009/8/1 | PHASE1 |
| NCT00990028 | Effect of Rosuvastatin on Cytokines After Traumatic Brain Injury | DRUG: Rosuvastatin\|DRUG: Placebo | COMPLETED | 2009/8/1 | PHASE1\|PHASE2 |
| NCT00983437 | Study to Evaluate the Safety, Tolerability, and Efficacy of Armodafinil as Treatment for Patients With Excessive Sleepiness Associated With Mild or Moderate Closed Traumatic Brain Injury | DRUG: Armodafinil | TERMINATED | 2009/8/31 | PHASE3 |
| NCT00973999 | Botulinum Toxin and Saliva Management in Tracheotomised Patients | DRUG: Botulinum Toxin | WITHDRAWN | 2009/9/1 | PHASE2 |
| NCT00908063 | Safety and Tolerability of Oxycyte in Patients With Traumatic Brain Injury (TBI) | DRUG: Oxycyte\|DRUG: Normal Saline | TERMINATED | 2009/10/1 | PHASE2 |
| NCT01000064 | Treatment of Traumatic Brain Injury (TBI)-Related Attention Deficits | DRUG: Vyvanse\|PROCEDURE: fMRI\|DRUG: Placebo | TERMINATED | 2009/10/1 | PHASE3 |
| NCT02012582 | Phase 2 a Study to Assess Safety and Pharmacokinetics of VAS203 in Patients With Traumatic Brain Injury | DRUG: VAS203 | COMPLETED | 2009/11/1 | PHASE2 |
| NCT01014403 | Delayed Versus Early Enoxaparin Prophylaxis After Traumatic Brain Injury (TBI) | DRUG: enoxaparin\|DRUG: placebo | COMPLETED | 2009/11/1 | PHASE1 |
| NCT01699308 | Growth Hormone and Brain Functioning After Traumatic Brain Injury | DRUG: Genotropin | COMPLETED | 2009/12/1 | PHASE1 |
| NCT01132703 | Safety Study of RP-1127 (Glyburide for Injection) in Healthy Volunteers | DRUG: Glyburide for Injection\|DRUG: Placebo | COMPLETED | 2010/1/7 | PHASE1 |
| NCT01058395 | Safety and Feasibility of Minocycline in the Treatment of Traumatic Brain Injury (TBI) | DRUG: Minocycline | COMPLETED | 2010/2/1 | PHASE1\|PHASE2 |
| NCT01013870 | Mission Connect Mild Traumatic Brain Injury (TBI) Integrated Clinical Protocol | DRUG: Atorvastatin\|DRUG: Placebo | TERMINATED | 2010/2/1 | PHASE2 |
| NCT00822900 | Progesterone for the Treatment of Traumatic Brain Injury III | DRUG: Progesterone\|DRUG: Placebo | TERMINATED | 2010/3/1 | PHASE3 |
| NCT01368432 | Lexapro for the Treatment of Traumatic Brain Injury (TBI) Depression & Other Psychiatric Conditions | DRUG: Escitalopram\|DRUG: Placebo | COMPLETED | 2010/4/1 | PHASE2 |
| NCT01111682 | Hypertonic Saline vs. Mannitol for Elevated Intercranial Pressure | DRUG: Mannitol\|DRUG: Hypertonic Saline | TERMINATED | 2010/4/1 | PHASE3 |
| NCT00987454 | Erythropoietin in Traumatic Brain Injury (EPO-TBI) | DRUG: Epoetin Alfa\|DRUG: Sodium Chloride 0.9% | COMPLETED | 2010/5/1 | PHASE3 |
| NCT01143064 | Efficacy and Safety Study of Intravenous Progesterone in Patients With Severe Traumatic Brain Injury | DRUG: Progesterone\|DRUG: Lipid emulsion without progesterone | COMPLETED | 2010/6/1 | PHASE3 |
| NCT01135862 | Platelet Administration To Patients With Traumatic Brain Injury Who Were Treated With Aspirin | DRUG: platelets | UNKNOWN | 2010/6/1 | PHASE2 |
| NCT01202110 | Early Propranolol After Traumatic Brain Injury: Phase II | DRUG: Propranolol | TERMINATED | 2010/6/1 | PHASE2 |
| NCT00761228 | Efficacy Study of NH001 in Vegetative State & Minimally Conscious State Following a Traumatic Brain Injury | DRUG: Apomorphine\|DRUG: Placebo | SUSPENDED | 2010/7/1 | PHASE2 |
| NCT01093261 | Corticosteroid Therapy for Glucocorticoid Insufficiency Related to Traumatic Brain Injury | DRUG: Placebo\|DRUG: Hydrocortisone Fludrocortisone | COMPLETED | 2010/8/1 | PHASE3 |
| NCT01201863 | Neuroendocrine Dysfunction in Traumatic Brain Injury: Effects of Testosterone Therapy | DRUG: Androgel (Testosterone Gel)\|OTHER: Placebo gel | COMPLETED | 2010/9/1 | PHASE4 |
| NCT01207050 | Effect of Rozerem on Sleep Among People With Traumatic Brain Injury | DRUG: Ramelteon\|DIETARY_SUPPLEMENT: Placebo | UNKNOWN | 2010/9/1 | PHASE4 |
| NCT01231139 | The Paracetamol AfteR Traumatic Brain InjurY Study | DRUG: Paracetamol\|DRUG: 0.9% Sodium Chloride Schedule: | COMPLETED | 2010/10/1 | PHASE2 |
| NCT00810940 | Controlled Trial of ABELADRUG200 in Closed, Severe Head Injury | DRUG: AbelaDrug200\|DRUG: mannitol | UNKNOWN | 2010/10/1 | PHASE1\|PHASE2 |
| NCT01239706 | Safety Study of Human Chorionic Gonadotropin (hCG) and Epoetin Alfa (EPO) in Traumatic Brain Injury: Dosing Tier 1 | DRUG: NTx 265 | UNKNOWN | 2010/11/1 | PHASE2 |
| NCT01212679 | Effects of Intranasal Nerve Growth Factor for Traumatic Brain Injury | DRUG: nerve growth factor\|DRUG: nomral saline | COMPLETED | 2010/12/1 | PHASE2 |
| NCT01306968 | Hyperbaric Oxygen Therapy (HBO2) for Persistent Post-concussive Symptoms After Mild Traumatic Brain Injury (mTBI) | DRUG: hyperbaric oxygen\|OTHER: sham hyperbaric air | COMPLETED | 2011/2/1 | PHASE2 |
| NCT01322009 | Overcoming Membrane Transporters to Improve CNS Drug Delivery - Improving Brain Antioxidants After Traumatic Brain Injury | DRUG: Probenecid and N-acetyl cysteine\|DRUG: Placebo | COMPLETED | 2011/3/1 | PHASE1\|PHASE2 |
| NCT01357421 | Effects of TT301 on Cytokine Levels Post Endotoxin Challenge | DRUG: TT301\|DRUG: Placebo | COMPLETED | 2011/5/1 | PHASE1 |
| NCT01343329 | Controlling Hyperadrenergic Activity in Neurologic Injury | DRUG: Esmolol\|DRUG: Propranolol | WITHDRAWN | 2011/7/1 | PHASE1\|PHASE2 |
| NCT01322048 | DASH After TBI Study: Decreasing Adrenergic or Sympathetic Hyperactivity After Traumatic Brain Injury | DRUG: IV Propranolol and Per Tube Clonidine\|DRUG: Placebo | COMPLETED | 2011/8/1 | PHASE2 |
| NCT01343862 | The Effect of Single Dose D-Cycloserine on Cognitive Outcome in Moderate Traumatic Brain Injury (TBI) Patients | DRUG: D-cycloserine\|DRUG: placebo | UNKNOWN | 2011/8/1 | PHASE2 |
| NCT01416948 | Cognitive REmediation After Trauma Exposure Trial = CREATE Trial | DRUG: Methylphenidate Hydrochloride 20 mg\|DRUG: Placebo Capsule\|DRUG: Galantamine 12 mg | TERMINATED | 2011/8/1 | PHASE2 |
| NCT01342549 | Treatment Strategy for Alcohol Use Disorders in Veterans With TBI | DRUG: Valproate\|DRUG: Naltrexone | COMPLETED | 2011/9/1 | PHASE3 |
| NCT01712477 | A Comparison of Propofol Versus Midazolam to Sedate Critically Brain Injury; Measurement of Cytokine Response and Assessment of Function | DRUG: Intravenous sedation using propofol\|DRUG: Intravenous sedation with midazolam | TERMINATED | 2011/11/1 | PHASE4 |
| NCT01397500 | Hormone Treatment in Growth Hormone and Testosterone Deficient Patients | DRUG: Genotropin\|DRUG: Testosterone undecannoate | TERMINATED | 2011/11/1 | PHASE2 |
| NCT01374633 | Sevoflurane Sedation on Intra Cranial Pressure in Traumatic Brain Injury Patients | DRUG: 1: Sevoflurane | TERMINATED | 2011/12/1 | PHASE2 |
| NCT01333488 | Mild Hypothermia and Supplemental Magnesium Sulfate Infusion in Severe Traumatic Brain Injury (TBI) Subjects | DEVICE: Arctic Sun\|DRUG: Magnesium Sulfate | TERMINATED | 2011/12/1 | PHASE2 |
| NCT01454154 | Glyburide (RP-1127) for Traumatic Brain Injury (TBI) | DRUG: Glyburide\|DRUG: Placebo | COMPLETED | 2011/12/17 | PHASE2 |
| NCT01470040 | Does Discontinuation of Aspirin Treatment Following Head Trauma Decrease the Incidence of Chronic Subdural Hematoma? | DRUG: discontinuation of aspirin therapy\|DRUG: continuation of aspirin therapy | UNKNOWN | 2012/2/1 | PHASE4 |
| NCT01420042 | Safety Study of NNZ-2566 in Healthy Subjects, Following Oral Administration | DRUG: NNZ-2566\|DRUG: Placebo | COMPLETED | 2012/2/1 | PHASE1 |
| NCT01591759 | A Pilot Trial of Citicoline in Individuals With Mild Traumatic Brain Injury (mTBI) | DRUG: Citicoline\|DRUG: Placebo | WITHDRAWN | 2012/4/1 | PHASE2 |
| NCT01402882 | Clinical Randomisation of an Antifibrinolytic in Significant Head Injury | DRUG: Tranexamic Acid | COMPLETED | 2012/7/1 | PHASE3 |
| NCT01605357 | Hypernatremia for the Prevention and Treatment of Cerebral Edema in Traumatic Brain Injury | DRUG: Induced, sustained hypernatremia using hypertonic saline\|DRUG: Standard of care (hypertonic saline and mannitol; serum sodium) | WITHDRAWN | 2012/7/1 | PHASE1\|PHASE2 |
| NCT01750268 | Topiramate Treatment of Hazardous and Harmful Alcohol Use in Veterans With TBI | BEHAVIORAL: Medical Management Counseling\|DRUG: Topiramate\|DRUG: Placebo | COMPLETED | 2012/11/1 | PHASE4 |
| NCT01670526 | Rivastigmine Patch in Veterans With Cognitive Impairment Following TBI | DRUG: Rivastigmine Transdermal Patch | COMPLETED | 2012/12/1 | PHASE3 |
| NCT02312635 | Augmentation of Cognitive Training in Children With TBI With D-Cyloserine | DRUG: D-Cycloserine\|BEHAVIORAL: Cogmed Working Memory Training | UNKNOWN | 2012/12/1 | EARLY_PHASE1 |
| NCT01762475 | Sildenafil for Cerebrovascular Dysfunction in Chronic Traumatic Brain Injury. | DRUG: Sildenafil | COMPLETED | 2013/1/1 | PHASE2 |
| NCT01366820 | Study of NNZ-2566 in Patients With Traumatic Brain Injury Under EFIC | DRUG: NNZ-2566\|DRUG: Placebo | COMPLETED | 2013/2/1 | PHASE2 |
| NCT01799941 | Safety, Tolerability and Effectiveness of Nuedexta in the Treatment of Pseudobulbar Affect (PBA) | DRUG: Nuedexta (DM 20 mg/Q 10 mg) | COMPLETED | 2013/2/1 | PHASE4 |
| NCT01815125 | Ondansetron for Pediatric Mild Traumatic Brain Injury | DRUG: Ondansetron\|DRUG: PLacebo | COMPLETED | 2013/3/1 | PHASE1 |
| NCT01847755 | Hyperbaric Treatment of Traumatic Brain Injury (TBI) | DRUG: Oxygen at 1.5 ATA (atmospheres absolute). | COMPLETED | 2013/4/1 | PHASE1\|PHASE2 |
| NCT01673828 | Allopregnanolone for the Treatment of Traumatic Brain Injury | DRUG: Allopregnanolone injection\|DRUG: Placebo injection | COMPLETED | 2013/4/1 | PHASE2 |
| NCT01825044 | Copenhagen Head Injury Ciclosporin (CHIC) Study | DRUG: NeuroSTAT 5 mg/kg/day\|DRUG: NeuroSTAT 10 mg/kg/day | COMPLETED | 2013/4/1 | PHASE2 |
| NCT01856270 | Amitriptyline to Prevent Headache After Traumatic Brain Injury | DRUG: Amitriptyline | COMPLETED | 2013/4/1 | PHASE2 |
| NCT01854385 | Sumatriptan as Treatment for Post-traumatic Headache | DRUG: Sumatriptan 100 mg | COMPLETED | 2013/6/1 | PHASE2 |
| NCT01948505 | Effect of IV Acetaminophen on Patients in the Neurocritical Care Unit | DRUG: Intravenous acetaminophen\|DRUG: Placebo for IV acetaminophen | COMPLETED | 2013/8/1 | PHASE4 |
| NCT01903525 | DHA For The Treatment of Pediatric Concussion Related to Sports Injury | DRUG: Docosahexaenoic acid (DHA)\|DRUG: Placebo | COMPLETED | 2013/9/1 | PHASE1 |
| NCT02255799 | Multicenter Evaluation of Memory Remediation After TBI With Donepezil | DRUG: Donepezil\|DRUG: Placebo | ACTIVE_NOT_RECRUITING | 2013/9/1 | PHASE3 |
| NCT01606111 | The CAPTAIN Trial: Cerebrolysin Asian Pacific Trial in Acute Brain Injury and Neurorecovery | DRUG: Cerebrolysin\|DRUG: 0.9% NaCl, saline | TERMINATED | 2013/9/1 | PHASE4 |
| NCT02071407 | A Clinical Trial of the Effect of Midazolam on the Cerebral Metabolism and Inflammatory Response in Patients With Moderate and Severe Traumatic Brain Injury | DRUG: Midazolam\|DRUG: Placebo | UNKNOWN | 2013/10/1 | PHASE4 |
| NCT01336413 | Neuroactive Steroids and Traumatic Brain Injury (TBI) in OEF/OIF Veterans | DRUG: Pregnenolone\|DRUG: Placebo | COMPLETED | 2013/10/1 | PHASE2 |
| NCT01933217 | Methylphenidate for Attention Problems After Pediatric TBI | DRUG: Methylphenidate\|DRUG: Placebo | COMPLETED | 2013/11/1 | PHASE4 |
| NCT01676311 | Effects of Huperzine A in Treatment of Moderate to Severe TBI | DRUG: Huperzine A\|DRUG: Placebo | TERMINATED | 2013/12/1 | PHASE2 |
| NCT01860404 | BCAA's in Concussion | DRUG: Branched Chain Amino Acids\|DRUG: Placebo solution | COMPLETED | 2014/1/1 | PHASE2 |
| NCT02015949 | The Relationship Between Traumatic Brain Injury and Dopamine (a Chemical in the Brain) | DRUG: Methylphenidate\|DRUG: Placebo | COMPLETED | 2014/2/1 | PHASE4 |
| NCT02091739 | Clinical Study to Investigate the Efficacy and Safety of Two Dose Levels of NT 201 Versus Placebo in Treating Chronic Troublesome Sialorrhea in Various Neurological Conditions | DRUG: IncobotulinumtoxinA (100 Units)\|DRUG: IncobotulinumtoxinA (75 Units)\|DRUG: Placebo | COMPLETED | 2014/4/1 | PHASE3 |
| NCT02116348 | Cerebrolysin Neural Repair Therapy in Children With Traumatic Brain Injury and Cerebral Palsy | DRUG: Cerebrolysin (Nerve growth factor) | UNKNOWN | 2014/4/1 | PHASE2 |
| NCT02089594 | Hyperbaric Oxygen Treatment to Treat Mild Traumatic Brain Injury (mTBI)/Persistent Post-Concussion Syndrome (PPCS) | DRUG: Hyperbaric Oxygen\|DRUG: No Hyperbaric Oxygen | UNKNOWN | 2014/5/1 | PHASE3 |
| NCT02160535 | Treatment of Chronic Post-Traumatic Headache With OnabotulinumtoxinA | DRUG: OnabotulinumtoxinA | TERMINATED | 2014/5/1 | PHASE3 |
| NCT02161055 | Intensive Versus Nonintensive Insulin Therapy for Hyperglycemia After Traumatic Brain Injury | DRUG: Insulin | UNKNOWN | 2014/6/1 | PHASE4 |
| NCT02321761 | Effect of Amantadine Administration on Spatial Functioning Following Traumatic Brain Injury | DRUG: Amantadine hydrochloride | UNKNOWN | 2014/6/1 | PHASE4 |
| NCT02225106 | Dopamine Receptor Imaging to Predict Response to Stimulant Therapy in Chronic TBI | DRUG: Methylphenidate | COMPLETED | 2014/8/6 | PHASE2 |
| NCT02226848 | Effect of Recombinant Erythropoietin on Numbers of Circulating Endothelial Progenitor Cells in People With Persistent Symptoms During the Subacute Period After Traumatic Brain Injury | DRUG: Erythropoeitin | WITHDRAWN | 2014/8/15 | PHASE2 |
| NCT02100150 | A Safety and Efficacy Study of NNZ-2566 in Patients With Mild Traumatic Brain Injury (mTBI) | DRUG: NNZ-2566\|DRUG: Placebo | TERMINATED | 2014/9/1 | PHASE2 |
| NCT02148783 | Dopamine Receptor Imaging to Predict Response to Stimulant Therapy in Chronic TBI | DRUG: methylphenidate\|DRUG: Placebo | TERMINATED | 2014/9/1 | PHASE2 |
| NCT02019810 | Impact of Cardiac Blood Flow on Cerebral Blood Flow in Patients With Severe Traumatic Brain Injury | DRUG: Norepinephrine\|DRUG: Dobutamine and norepinephrine | UNKNOWN | 2014/10/1 | PHASE2 |
| NCT02232347 | Ketamine and Glutamate After Brain Injury : a Microdialysis Study | DRUG: Ketamine\|DRUG: Sufentanil | UNKNOWN | 2014/10/1 | PHASE2 |
| NCT03119948 | Partial Blocks of Rectus Femoris and Soleus With Botulinum Toxin Type A (Xeomin庐) to Improve Gait in Hemiparesis | DRUG: Placebo injection\|DRUG: Botulinum toxin injection\|DRUG: Placebo injection and botulinum toxin injection | UNKNOWN | 2014/12/1 | PHASE2 |
| NCT02270736 | Clinical Study to Investigate the Efficacy and Safety of NT 201 Compared to Placebo in the Treatment of Chronic Troublesome Drooling Associated With Neurological Disorders and/or Intellectual Disability | DRUG: NT 201 Placebo\|DRUG: NT 201 | COMPLETED | 2015/2/9 | PHASE3 |
| NCT02404779 | Treatment of Intracranial Hypertension of Severe Tramatic Brain Injured Patients. Physiopathologic Effects of Neuromuscular Blocking Agents | DRUG: cisatracurium besilate\|OTHER: Placebo | UNKNOWN | 2015/3/19 | PHASE4 |
| NCT01990768 | Prehospital Tranexamic Acid Use for Traumatic Brain Injury | DRUG: 1 gram Tranexamic Acid (TXA)\|DRUG: 2 grams TXA\|DRUG: 0.9% Sodium Chloride injectable | COMPLETED | 2015/5/1 | PHASE2 |
| NCT02443142 | Ibuprofen Versus Acetaminophen for Treatment of Mild Traumatic Brain Injury | DRUG: Ibuprofen\|DRUG: Acetaminophen | WITHDRAWN | 2015/5/1 | PHASE2 |
| NCT02501941 | Spreading Depolarization and Ketamine Suppression | DRUG: ketamine | COMPLETED | 2015/7/1 | PHASE1 |
| NCT02652598 | Evaluate the Effects of Tolcapone on Cognitive and Behavioral Dysfunction in Patients With BI and NCD | DRUG: Tolcapone | UNKNOWN | 2015/11/1 | PHASE2 |
| NCT02645552 | Pre-hospital Administration of Tranexamic Acid for Moderate and Severe Traumatic Brain Injury | DRUG: Tranexamic Acid\|OTHER: Sodium chloride | UNKNOWN | 2016/1/1 | PHASE3 |
| NCT02957331 | Beta Blockade in in Traumatic Brain Injury | DRUG: Propranolol | COMPLETED | 2016/1/1 | PHASE4 |
| NCT02266329 | Chronic Postconcussive Headache: A Placebo-Controlled Treatment Trial of Prazosin | DRUG: prazosin hydrochloride\|DRUG: placebo | COMPLETED | 2016/1/4 | PHASE1\|PHASE2 |
| NCT02957123 | Intranasal Inhalations of Bioactive Factors Produced by M2 Macrophages in Patients With Organic Brain Syndrome | DRUG: Intranasal auto-M2-BFs | COMPLETED | 2016/3/1 | PHASE1\|PHASE2 |
| NCT02794168 | Efficacy of VAS203 (Ronopterin) in Patients With Moderate and Severe Traumatic Brain Injury | DRUG: VAS203 | COMPLETED | 2016/6/1 | PHASE3 |
| NCT02791945 | N-acetylcysteine Treatment of Alcohol Use Disorder In Veterans With TBI | BEHAVIORAL: Medical Management Counseling\|DRUG: N-acetylcysteine\|DRUG: Placebo | COMPLETED | 2016/8/1 | PHASE2 |
| NCT03401515 | Using Propranolol in Traumatic Brain Injury to Reduce Sympathetic Storm Phenomenon | DRUG: Propranolol Hydrochloride 1 MG/ML\|OTHER: Normal saline | COMPLETED | 2016/10/1 | PHASE4 |
| NCT02845349 | Vortioxetine for the Treatment of Major Depression and Co-morbidities After Traumatic Brain Injury (TBI) | DRUG: Vortioxetine\|DRUG: Placebo | WITHDRAWN | 2016/10/1 | PHASE3 |
| NCT02931474 | Impact of GHRH on Sleep Promotion and Endocrine Regulation in Service Members Who Sustained a Traumatic Brain Injury and Have Current Insomnia | DRUG: Tesamorelin\|OTHER: Placebo | WITHDRAWN | 2016/10/6 | PHASE2 |
| NCT02712996 | Treatment of Traumatic Brain Injury (TBI)-Related Attention Deficits in Children | DRUG: Lisdexamfetamine\|DRUG: Placebo | COMPLETED | 2017/2/6 | PHASE4 |
| NCT03095066 | Study to Assess the Efficacy, Safety, and Tolerability of AVP-786 for the Treatment of Neurobehavioral Disinhibition Including Aggression, Agitation, and Irritability in Participants With Traumatic Brain Injury | DRUG: Placebo\|DRUG: AVP-786-28\|DRUG: AVP-786-42.63 | COMPLETED | 2017/5/30 | PHASE2 |
| NCT03054285 | Does Short-Term Anti-Seizure Prophylaxis After Traumatic Brain Injury Decrease Seizure Rates? | DRUG: Levetiracetam | UNKNOWN | 2017/7/1 | PHASE4 |
| NCT03273062 | A Trial Evaluating Effects of COMT Inhibition in Patients With Acquired Brain Injury | DRUG: Tolcapone 200 MG\|OTHER: Placebo | UNKNOWN | 2017/7/20 | PHASE2 |
| NCT02496975 | Traumatic Brain Injury and Effects of Acute Cyclosporine A | DRUG: Cyclosporine A\|OTHER: Placebo | WITHDRAWN | 2017/8/7 | PHASE1\|PHASE2 |
| NCT03475693 | A Cohort Study Evaluating the Efficacy of PO Magnesium in the Treatment of Acute Traumatic Brain Injury in Adolescents | DRUG: Magnesium Oxide\|DRUG: Acetaminophen\|DRUG: Zofran ODT 4 MG Disintegrating Oral Tablet | COMPLETED | 2017/9/1 | EARLY_PHASE1 |
| NCT03345550 | OPTIMA-TBI Pilot Study | DRUG: Omega-3 Polyunsaturated Fatty Acids (Fish Oil 1000 mg (contains 500 mg DHA & 100 mg EPA)) or placebo capsules.\|DRUG: Placebo - Cap | TERMINATED | 2017/9/12 | PHASE2 |
| NCT02997371 | IL-1ra Dose-range Study for Moderate-to-severe TBI Patients | DRUG: Anakinra Prefilled Syringe\|DRUG: Isotonic saline | UNKNOWN | 2017/10/1 | PHASE2 |
| NCT03143751 | Continuous Hyperosomolar Therapy for Traumatic Brain-injured Patients | DRUG: NaCl20% (Continuous hyperosmolar therapy) | COMPLETED | 2017/10/31 | PHASE3 |
| NCT03430817 | Citicholine-Amantadine Trial in Traumatic Brain Injury | DRUG: Citicholine\|DRUG: Amantadine | UNKNOWN | 2017/12/7 | PHASE4 |
| NCT01048138 | Use of Biperiden for the Prevention of Post-traumatic Epilepsy | DRUG: Biperiden Lactate\|DRUG: Placebo | TERMINATED | 2018/1/31 | PHASE3 |
| NCT03417492 | Cerebrovascular Reactivity in American Football Players | DRUG: Sildenafil Citrate | TERMINATED | 2018/3/1 | PHASE1 |
| NCT02407028 | Hyperbaric Oxygen Brain Injury Treatment Trial | DRUG: Hyperbaric oxygen (1.5 ATA, no NBH)\|DRUG: Hyperbaric oxygen (2.0 ATA, no NBH)\|DRUG: Hyperbaric oxygen (2.5 ATA, no NBH)\|DRUG: Hyperbaric oxygen (1.5 ATA + NBH)\|DRUG: Hyperbaric oxygen (2.0 ATA + NBH)\|DRUG: Hyperbaric oxygen (2.5 ATA + NBH)\|DRUG: Normobaric Hyperoxia (NBH)\|OTHER: Usual Care | RECRUITING | 2018/6/25 | PHASE2 |
| NCT03598140 | Sildenafil Treatment for Mild TBI | DRUG: Sildenafil Citrate\|DRUG: Placebo oral capsule | TERMINATED | 2018/7/25 | PHASE2 |
| NCT03554265 | Brain and Gut Plasticity in Mild TBI or Post-acute COVID Syndrome Following Growth Hormone Therapy | DRUG: Somatropin | COMPLETED | 2018/8/6 | PHASE3 |
| NCT04487275 | MLC901 for Moderate to Severe Traumatic Brain Injury (Specified Drug Code) | DRUG: MLC901 or Placebo | UNKNOWN | 2018/8/20 | PHASE4 |
| NCT03496545 | Efficacy of Bromocriptine For Fever Reduction in Acute Neurologic Injury | DRUG: Bromocriptine 5 MG\|DRUG: Acetaminophen 650 MG | COMPLETED | 2018/11/30 | PHASE1\|PHASE2 |
| NCT03944447 | Outcomes Mandate National Integration With Cannabis as Medicine | DRUG: Cannabis, Medical\|DEVICE: RYAH-Medtech Inhaler | RECRUITING | 2018/12/1 | PHASE2 |
| NCT03260569 | Inhaled Nitric Oxide in Brain Injury | DRUG: Inhaled Nitric Oxide\|DRUG: Placebo | COMPLETED | 2018/12/12 | PHASE3 |
| NCT03845322 | Curcumin/Turmeric as a Treatment for Patients With Subdural Hematomas Recurrence | DRUG: Curcumin Pill\|DRUG: Placebo pills | WITHDRAWN | 2018/12/18 | EARLY_PHASE1 |
| NCT04280965 | Quetiapine Augmentation of PE Therapy for the Treatment of Co-occurring PTSD and Mild Traumatic Brain Injury | DRUG: Quetiapine Fumarate\|OTHER: Standard of care meducations | COMPLETED | 2019/2/1 | EARLY_PHASE1 |
| NCT02840097 | Traumatic Injury Clinical Trial Evaluating Tranexamic Acid in Children: A Pilot and Feasibility Study | DRUG: Tranexamic Acid\|DRUG: Placebo | COMPLETED | 2019/3/4 | PHASE2 |
| NCT03974360 | Efficacy and Tolerability of Erenumab in the Prophylactic Treatment of Persistent Post-Traumatic Headache | DRUG: AMG 334 | COMPLETED | 2019/4/5 | PHASE2 |
| NCT04646876 | Mgso4 as Neuroprotective in Post Traumatic Brain Injury | DRUG: Magnesium Sulfate\|DRUG: Placebo | COMPLETED | 2019/5/2 | PHASE3 |
| NCT04291066 | Prospective Analysis of the Use of N-Acetylcysteine and Vitamins in the Treatment of TBI in Geriatric Patients | DRUG: N-Acetyl cysteine | COMPLETED | 2019/9/1 | PHASE2 |
| NCT03992404 | Study to Compare the Efficacy and Safety of NT 201 (Botulinum Toxin) With Placebo for the Treatment of Lower Limb Spasticity Caused by Stroke or Traumatic Brain Injury | DRUG: NT 201\|DRUG: Placebo | RECRUITING | 2019/9/16 | PHASE3 |
| NCT03954041 | A Study to Evaluate the Efficacy and Safety of BIIB093 in Participants With Brain Contusion | DRUG: BIIB093\|DRUG: Placebo | TERMINATED | 2019/10/6 | PHASE2 |
| NCT04239755 | Doxycycline in Acute Traumatic Brain Injury | DRUG: Doxycycline 100 MG Oral Tablet\| OTHER: placebo | COMPLETED | 2020/2/1 | PHASE4 |
| NCT04226365 | Pediatric Concussion Outcomes | DRUG: Nortriptyline\| DRUG: Placebo | ACTIVE_NOT_RECRUITING | 2020/2/15 | PHASE4 |
| NCT04499755 | Efficacy of Nucleo CMP Forte in Traumatic Brain Injury in Pediatrics | DRUG: Nucleo CMP Forte | RECRUITING | 2020/6/1 | PHASE3 |
| NCT04426487 | Effect of Progesterone Therapy on Traumatic Subarachinoid Haemorrhage on Clinical Outcome and Resistive Vasculer Indices of Middle Cerebral Artery Transcranial Doppler | DRUG: Progesterone | UNKNOWN | 2020/6/20 | EARLY_PHASE1 |
| NCT04303065 | Dexamethasone for the Treatment of Vasogenic Pericontusional Edema. | DRUG: Dexamethasone Oral\| DRUG: Placebo oral tablet | RECRUITING | 2020/7/24 | PHASE3 |
| NCT04400266 | Buspirone and Melatonin for Depression Following Traumatic Brain Injury | DRUG: B+MEL | UNKNOWN | 2020/8/1 | PHASE4 |
| NCT03814356 | Stimulant Therapy Targeted to Individualized Connectivity Maps to Promote ReACTivation of Consciousness | DRUG: Methylphenidate | RECRUITING | 2020/8/24 | PHASE1 |
| NCT03626727 | Evaluation of the Efficacy of Sodium Oxybate (Xyrem庐) in Treatment of Post-traumatic Narcolepsy and Post-traumatic Hypersomnia | DRUG: Sodium Oxybate Oral Solution [Xyrem] | WITHDRAWN | 2020/9/1 | EARLY_PHASE1 |
| NCT02776488 | Exogenous Sodium Lactate Infusion in Traumatic Brain Injury (ELI-TBI) | DRUG: Sodium Lactate\| DRUG: Placebo | WITHDRAWN | 2020/9/1 | PHASE2 |
| NCT04527289 | Impact of Amantadine on Traumatic Brain Injury | DRUG: Amantadine (100mg) as add on therapy.\|DRUG: Placebo | COMPLETED | 2020/9/30 | PHASE4 |
| NCT04631484 | International Trial of Efficacy of Cytoflavin in Head Trauma | DRUG: Cytoflavin ((Inosine + Nicotinamide + Riboflavin + Succinic Acid)\|DRUG: Placebo | RECRUITING | 2020/10/8 | PHASE3 |
| NCT04558346 | Ghrelin (OXE--103) for Acute Concussion Management | DRUG: Ghrelin (OXE-103)\|DRUG: Placebo | COMPLETED | 2020/10/20 | PHASE2 |
| NCT04588311 | ErythroPOietin Alfa to Prevent Mortality and Reduce Severe Disability in Critically Ill TRAUMA Patients | DRUG: Epoetin Alfa 40000 UNT/ML\|DRUG: Sodium Chloride 0.9% | RECRUITING | 2020/11/9 | PHASE3 |
| NCT04686344 | Point of Care Optic Nerve Sheath Ultrasound to Assess Intracranial Pressure | DRUG: intermittent boluses of Hypertonic saline\|DRUG: continuous infusion of Hypertonic saline | COMPLETED | 2020/12/21 | EARLY_PHASE1 |
| NCT04508244 | Beta Blocker Use In Traumatic Brain Injury Based On The High-Sensitive Troponin T Status | DRUG: Placebo\|DRUG: Propranolol | RECRUITING | 2020/12/29 | PHASE4 |
| NCT04744051 | ATCell鈩?Expanded Autologous, Adipose-Derived Mesenchymal Stem Cells Deployed Via Intravenous Infusion | DRUG: 50 Adipose Derived Stem Cell Infusion\|DRUG: 150 Adipose Derived Stem Cell Infusion\|DRUG: 300 Adipose Derived Stem Cell Infusion\|DRUG: Placebo Infusion | ACTIVE_NOT_RECRUITING | 2021/2/1 | PHASE1 |
| NCT04489160 | Complement Inhibition: Attacking the Overshooting Inflammation @Fter Traumatic Brain Injury | DRUG: C1 Inhibitor, Human\|DRUG: Placebo | RECRUITING | 2021/2/25 | PHASE2 |
| NCT04521881 | Clinical Randomisation of an Anti-fibrinolytic in Symptomatic Mild Head Injury in Older Adults | DRUG: Tranexamic Acid 500 MG | RECRUITING | 2021/4/18 | PHASE3 |
| NCT04550377 | Cannabidiol as a Treatment for PTSD and PTSD Comorbid With TBI | DRUG: Cannabidiol\|DRUG: Placebo | RECRUITING | 2021/5/26 | PHASE2 |
| NCT05958277 | Vitamin B12 vs B3 for Nerve Regeneration and Functional Recovery After Pediatric Traumatic Brain Injury | DRUG: Vitamin B3\|DRUG: Vitamin B12 | COMPLETED | 2021/6/1 | PHASE3 |
| NCT05033444 | A First in Human Study of the Safety, Tolerability and Pharmacokinetics of PRV-002 in Healthy Volunteers | DRUG: PRV-002\|DRUG: Placebo | UNKNOWN | 2021/9/17 | PHASE1 |
| NCT05148403 | To Evaluate the Effect of Glibenclamide in Reducing Brain Edema of TBI | DRUG: glibenclamide | UNKNOWN | 2021/10/22 | PHASE1\|PHASE2 |
| NCT05058677 | Aerosolized Endotracheal Lidocaine to Avoid Intracranial Pressure Spikes in Patients With Severe Traumatic Brain Injury | DRUG: aerosolized 2% lidocaine (20mg/ml)\|DRUG: instilled 0.9% sodium chloride (NS)\|DRUG: instilled 2% lidocaine solution\|PROCEDURE: Endotracheal Suctioning (ETS) | RECRUITING | 2021/11/9 | PHASE4 |
| NCT04815967 | Efficacy and Safety Study of MYOBLOC庐 in the Treatment of Adult Upper Limb Spasticity | DRUG: Phase 2; Low Dose MYOBLOC\|DRUG: Phase 2; High Dose MYOBLOC\|DRUG: Phase 2; Placebo\|DRUG: Phase 3; MYOBLOC\|DRUG: Phase 3; Placebo | ACTIVE_NOT_RECRUITING | 2021/11/16 | PHASE2\|PHASE3 |
| NCT05131867 | Management of Cerebral Vascular Spasm in Posttraumatic Subarachnoid Hemorrhage Using Combination Therapy | DRUG: nimodipine\|DRUG: Oral Nimodipine and milrinone | COMPLETED | 2021/11/24 | PHASE2 |
| NCT05323149 | Tranexamic Acid in Traumatic Brain Injury | OTHER: Saline\|DRUG: Tranexamic acid | RECRUITING | 2022/5/18 | PHASE3 |
| NCT05173818 | Hyperbaric Oxygen Effects on Persistent Post-concussive Symptoms | DRUG: Hyperbaric oxygen at 1.5 ATA\|DRUG: Sham treatment | ACTIVE_NOT_RECRUITING | 2022/6/28 | PHASE2 |
| NCT05049057 | Treatment of Acute PTH With a CGRP Receptor mAb in Military Service Members and Civilians With mTBI | DRUG: Erenumab 140 Mg/mL Subcutaneous Solution\|DRUG: Placebo | RECRUITING | 2022/7/19 | PHASE2 |
| NCT05569993 | Glutamine and Traumatic Brain Injury | DRUG: Glutamine\|OTHER: Placcebo | NOT_YET_RECRUITING | 2022/10/30 | EARLY_PHASE1 |
| NCT05660356 | Growth Hormone Deficiency in Mild Traumatic Brain Injury | DRUG: Growth Hormone\|DRUG: Placebo | NOT_YET_RECRUITING | 2023/2/1 | EARLY_PHASE1 |
| NCT04731974 | Acute Concussion and Melatonin | DRUG: Melatonin 3 MG\|OTHER: Placebo | NOT_YET_RECRUITING | 2023/3/1 | PHASE2 |
| NCT05632627 | Cannabinoids and Traumatic Brain Injury: A Randomized, Placebo Controlled Trial | DRUG: Cannabidiol\|DRUG: Placebo | RECRUITING | 2023/4/11 | PHASE2 |
| NCT05671692 | Novel Intervention for Chronic Complex TBI in OEF/OIF/OND Veterans | DRUG: Pregnenolone\|DRUG: Placebo | NOT_YET_RECRUITING | 2023/6/1 | PHASE2 |
| NCT05970575 | Insulin-sensitizing NE3107 in Improving Sleep and Fatigue in Subjects With Traumatic Brain Injury | DRUG: NE3107 | NOT_YET_RECRUITING | 2023/8/21 | PHASE2 |
| NCT05095857 | The Anesthetic Ketamine as Treatment for Patients With Severe Acute Brain Injury | DRUG: S-ketamine\|OTHER: Isotonic saline (placebo) | RECRUITING | 2023/9/15 | PHASE4 |
| NCT06163833 | Mesenchymal Stromal Cells for Traumatic Brain Injury | DRUG: Mesenchymal stromal cell low dosage-80*10^6 cells\|DRUG: Mesenchymal stromal cell low dosage-160*10^6 cells\|OTHER: Placebo-storage solution | RECRUITING | 2023/9/19 | PHASE2 |
| NCT06065046 | Baricitinib for Moderate and Severe Traumatic Intracerebral Hemorrhage/Contusions | DRUG: Baricitinib 4 MG\|OTHER: Standard treatment | NOT_YET_RECRUITING | 2023/10/1 | PHASE2 |
| NCT06071949 | Efficacy and Tolerability of AP707 in Patients With Chronic Pain Due to Central Neuropathy of Any Genesis | DRUG: Adezunap\|DRUG: Placebo | NOT_YET_RECRUITING | 2023/10/15 | PHASE3 |
| NCT06072001 | Long Term Efficacy and Tolerability of AP707 in Patients With Chronic Pain Due to Central Neuropathy of Any Genesis | DRUG: Adezunap (AP707)\|DRUG: Placebo | NOT_YET_RECRUITING | 2023/10/15 | PHASE3 |
| NCT06062628 | Ketamine in Severe Traumatic Brain Injury | DRUG: Ketamine Hydrochloride | NOT_YET_RECRUITING | 2023/11/1 | EARLY_PHASE1 |
| NCT06081283 | Antiseizure Medication in Seizure Networks at Early Acute Brain Injury | DRUG: Phenobarbital Sodium Injection\|DRUG: Levetiracetam\|DRUG: Lacosamide Injectable Product\|DRUG: Valproate Sodium\|DRUG: Phosphenytoin | RECRUITING | 2023/11/20 | PHASE4 |
| NCT06096415 | Safety and Efficacy of ABX-101 in Participants Aged 18 to 50 Years of Age With Moderate to Severe Traumatic Brain Injury | DRUG: ABX-101 1mg\|DRUG: ABX-101 2mg | NOT_YET_RECRUITING | 2023/12/1 | PHASE2 |
| NCT05782244 | Sildenafil for Microvasculopathy in Chronic TBI | DRUG: Sildenafil Citrate low dose\|DRUG: Placebo\|DRUG: Sildenafil medium dose\|DRUG: Sildenafil high dose | NOT_YET_RECRUITING | 2023/12/15 | PHASE2 |
